# A new angiographic scoring for grading the difficulty of recanalization for symptomatic non-acute middle cerebral artery occlusions

**DOI:** 10.3389/fnins.2024.1398749

**Published:** 2024-10-08

**Authors:** Jie Cao, Xucheng Zhu, Sheng Liu, Yunfeng Zhang, Congguo Yin, Chongke Zhong, Yi Mo, Jinggang Xuan, Ronghua Chen, Chun Zhou, Guoxiang Huang, Wenqing Xia, Wei Xing, Ya Peng

**Affiliations:** ^1^Department of Neurosurgery, The Third Affiliated Hospital of Soochow University, Changzhou, China; ^2^Department of Interventional Radiology, The First Affiliated Hospital of Nanjing Medical University, Nanjing, China; ^3^Neurointervention Center, The Affiliated Hospital of Nantong University, Nantong, China; ^4^Department of Neurology, Affiliated Hangzhou First People’s Hospital, Zhejiang University School of Medical, Hangzhou, China; ^5^Department of Epidemiology, School of Public Health, Suzhou Medical College of Soochow University, Suzhou, China; ^6^Department of Radiology, The Third Affiliated Hospital of Soochow University, Changzhou, China

**Keywords:** symptomatic non-acute middle cerebral artery occlusion, endovascular treatment, recanalization, angiographic scoring, atherosclerosis, balloon dilation, stent

## Abstract

**Background:**

Endovascular recanalization is a feasible option for treating symptomatic non-acute middle cerebral artery occlusion (MCAO) patients. Hence, we aimed to establish a new angiographic scoring to grade the recanalization difficulty of MCAO to determine the suitable patients for endovascular treatment.

**Methods:**

We retrospectively analyzed a total of 113 consecutive recurrent symptomatic non-acute MCAO patients who underwent endovascular recanalization from July 2015 to August 2021 in four Chinese comprehensive stroke centers. All patients were reappraised using a new angiographic scoring based on the stump morphology, the MCA occlusion length, MCA bend, and the distal vascular bed of MCAO. We used the final results to establish the patients’ outcomes.

**Results:**

The total successful recanalization and perioperative complication rates were 83.2% (94/113) and 15.9% (18/113), respectively. No deaths occurred within 30 days. Moreover, 96.9, 90, 87.5, 52.6, and 50% of the patients achieved recanalization with scores of 0, 1, 2, 3, and 4 (*p <* 0.001), respectively. However, the perioperative complication rate showed the opposite trend. (3.1% vs. 7.5% vs. 6.3% vs. 52.6% vs. 50%; *p* < 0.001). The median time of successful microwire crossing of the occlusion lesion (TMO) in the score 0 group was shorter than the other groups (2 min, 9 min, 8.5 min, 14 min, and 20 min; *p* < 0.001). When a score of 2 was used as the optimal cut-off point, the sensitivity and specificity were 86.2 and 63.2%, respectively.

**Conclusion:**

The new angiographic scoring can effectively predict the successful recanalization rate, perioperative complication rate, and TMO of endovascular recanalization for non-acute MCAO. It can also be used as an effective clinical evaluation tool to determine the suitable non-acute MCAO patients for recanalization, especially with a score ≤ 2.

## 1 Introduction

Intracranial artery occlusive disease (IAOD) is the primary cause of stroke worldwide (Smith et al., 2009; Aghaebrahim et al., 2014). It can cause chronic cognitive dysfunction, thereby leading to a poor quality of life (Yamauchi et al., 2013; Zheng et al., 2019). In China, approximately 33–50% of strokes and 50% of transient ischemic attacks (TIAs) are attributed to IAOD (Wong, 2006). Moreover, patients with intracranial artery occlusion (IAO) report the highest incidence of recurrent stroke, i.e., 7.27% during the 1-year follow-up, while 5.16% of recurrent strokes occurred in those patients with 70–99% intracranial artery stenosis. Additionally, 3.82% of strokes occurred in the 50% ~ 69% intracranial artery stenosis group (Wang et al., 2014). A symptomatic non-acute IAO occurring 24 h after the symptom onset is a special IAOD subtype. These patients display a higher incidence of stroke recurrence despite aggressive medical therapy (AMT) and result in neurologic deficits, especially in hypoperfusion patients (Kuroda et al., 2001; Yamauchi et al., 2013; Shin et al., 2017). However, it was suggested that middle cerebral artery occlusion (MCAO) had the highest incidence among all IAODs in the CICAS study (Wang et al., 2014).

To date, the optimal treatment of symptomatic non-acute MCAO, especially in progressive stroke patients, is uncertain. Extracranial-intracranial (EC-IC) artery bypass surgery is not effective in symptomatic non-acute MCAO cases for preventing recurrent strokes (Bambakidis and Chowdhry, 2010; Powers et al., 2011). Considering the higher recurrent stroke rate in these patients reported in several studies (Yamauchi et al., 1996; Kuroda et al., 2001; Yamauchi et al., 2013), exploring more appropriate treatments for symptomatic non-acute MCAO becomes mandatory. Recently, few clinical studies have verified the feasibility and safety of endovascular recanalization for symptomatic non-acute MCAO (Ma et al., 2013; Aghaebrahim et al., 2014; Zhang et al., 2018; Zheng et al., 2019; Gao et al., 2021). However, many physicians do not attempt recanalization of the middle cerebral artery (MCA) owing to technical difficulties, lower recanalization rates, and perioperative complications. Gao et al. (2021) indicated that patients with a classification Type I MCA occlusion displayed a higher technical success rate (95.5%) and a lower perioperative complication rate (4.5%), which might be attributed to better preoperative assessment. However, this classification system might be too simple because preoperative angiography can often provide more clinical data and predictions (Gao et al., 2021). Thus, this study aimed to establish a new angiographic scoring to predict successful recanalization rate and to evaluate the technical drawbacks of recanalizing MCA in symptomatic non-acute MCAO patients. We also aspired to explore the suitable patients for this treatment and report our preliminary clinical results.

## 2 Materials and methods

### 2.1 Study population

Being a retrospective study, we reviewed the data of 113 consecutive patients with recurrent symptomatic non-acute MCAO treated with endovascular recanalization in four Chinese comprehensive stroke centers from July 2015 to December 2022.

The inclusion criteria were: (1) Patients with a non-acute MCA M1 segment occlusion (stroke symptoms beyond 24 h) and were diagnosed by either computed tomography angiography (CTA) or magnetic resonance angiography (MRA), as well as confirmed by digital subtraction angiography (DSA); (2) Those with atherosclerosis as the main etiological factor and were identified by high-resolution vessel wall imaging (HRVW); (3) Patients with recurrent TIA, stroke, increased neurological deficit, and consistent symptoms despite AMT (double antiplatelet therapy plus statins and risk factor management); (4) Those with hypoperfusion in the occluded MCA territory confirmed by a perfusion CT (CTP), and (5) Those having modified Rankin Scale (mRS) ≤3 before endovascular recanalization.

The exclusion criteria were: (1) Patients with non-atherosclerotic MCAO, such as vasculitis, moyamoya syndrome, or arterial dissection; (2) Those with underlying diseases such as cardiopulmonary insufficiency and malignant tumors that make them unsuitable for general anesthesia; (3) Patients with concomitant intracranial aneurysms or any bleeding disorder; (4) Presence of a large infarction core, defined as an Alberta Stroke Program Early CT Score (ASPECTS) <6 or a core infarct area > 70 mL; (5) no recurrent ischemic events after AMT, and (6) Absence of hypoperfusion in the occluded MCA territory, confirmed by CTP.

All patients (or their relatives) signed informed consent forms. This study was approved by the Ethics Committee of the Hospital [(2021) Section No. 134].

### 2.2 Angiographic scoring for symptomatic non-acute MCAO

**Figure 1:**
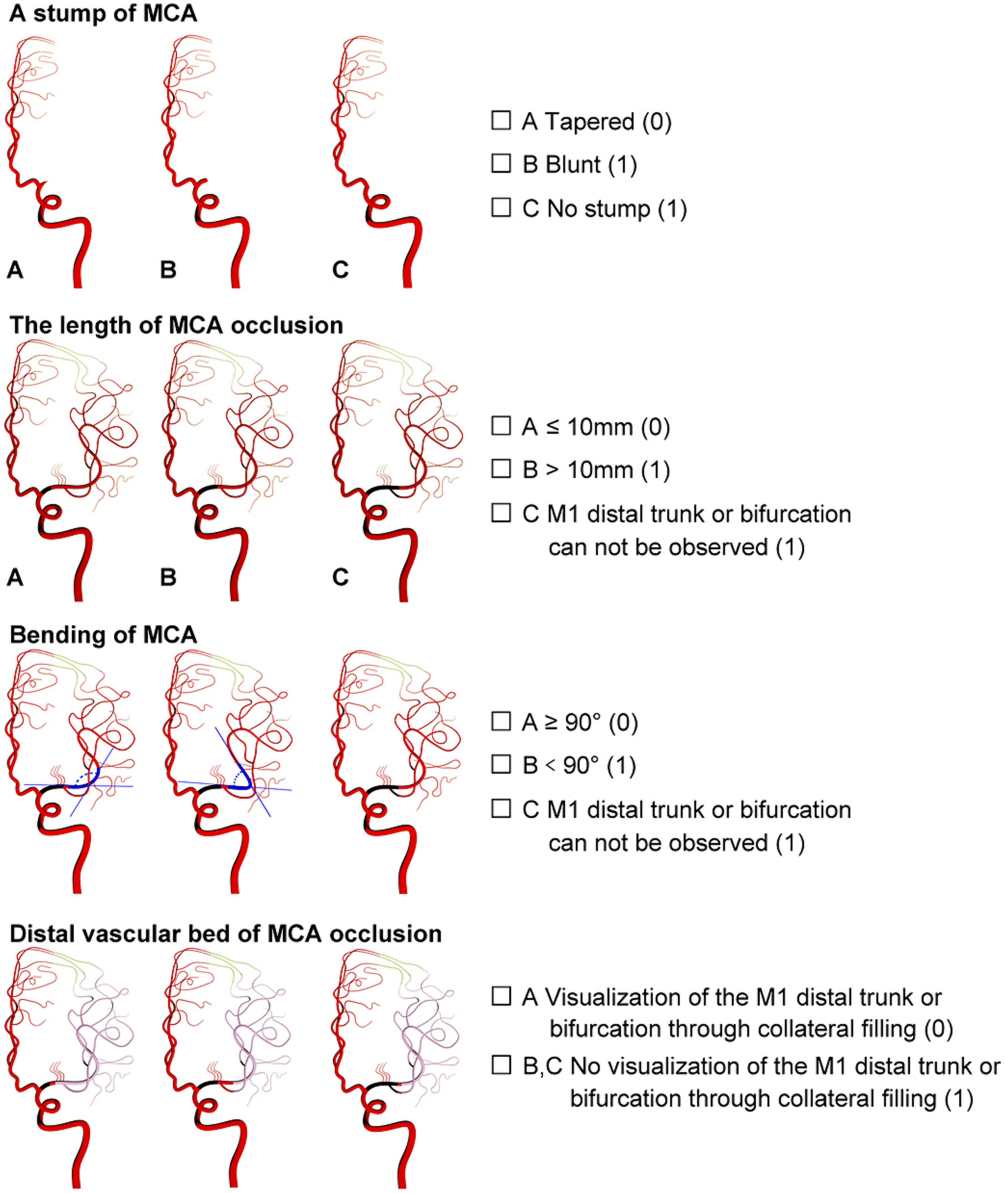
Angiographic scoring for symptomatic non-acute MCAO (MCAO-SEED). It consists of four parts: an MCA stump, the MCA occlusion length, the MCA bend, and the distal vascular bed of MCAO.

The new angiographic scoring for symptomatic non-acute MCAO was assessed by DSA with emphasis on the late retrograde or anterograde opacification of the M1 distal trunk, bifurcation, or M2 branch reconstruction via the leptomeningeal collateral pathway or/and basal perforating arteries. Furthermore, angiographic visualization of the M1 distal bifurcation was defined as the merging of at least two main MCA branches through collateral retrograde or anterograde fillings. The new scoring consisted of four parts and was named MCAO-SEED (Figure 1).

#### 2.2.1 S means MCA stump

An MCA stump was present if there was contrast filling within the initial MCA segment after it bifurcated from the ICA, proximally to the occluded segment. Angiographic morphology of the stump was designated “tapered” if the occluded segment ended in a funnel-shaped form or “blunt” if it did not. A “tapered” stump was counted as 0 points, while a “blunt” stump was counted as 1 point. Absence of stump was classified as a “blunt” stump and was given 1 point.

#### 2.2.2 First E means MCA occlusion length

The MCA occlusion length was defined as the distance between the proximal occlusion site and the M1 distal trunk; however, the bifurcation was reconstituted via the collateral vessels. An occlusion length ≤ 10 mm was counted as 0 points, while an occlusion length > 10 mm was counted as 1 point. Additionally, 1 point was allotted to a length of >10 mm or if the M1 distal trunk or bifurcation could not be observed.

#### 2.2.3 Second E means MCA bending

MCA bending was described as the angle between the MCA segment and the main MCA trunk, which was the branch where the microguide wire most likely entered the anteroposterior (AP) view. Angles ≥90° and < 90° were counted as 0 and 1 points, respectively. If the M1 distal trunk or bifurcation was not visible, a score of 1 point was allocated.

#### 2.2.4 D means distal MCAO vascular bed

Distal MCAO vascular bed: A good vascular bed was established as the visualization of the M1 distal trunk or bifurcation through the collateral retrograde or anterograde fillings. A good vascular bed was given 0 points; otherwise, it was counted as 1 point. One point was also designated when there was no visualization of the M1 distal trunk or bifurcation through the collateral retrograde or anterograde fillings.

### 2.3 Endovascular recanalization procedure

**Figure 2:**
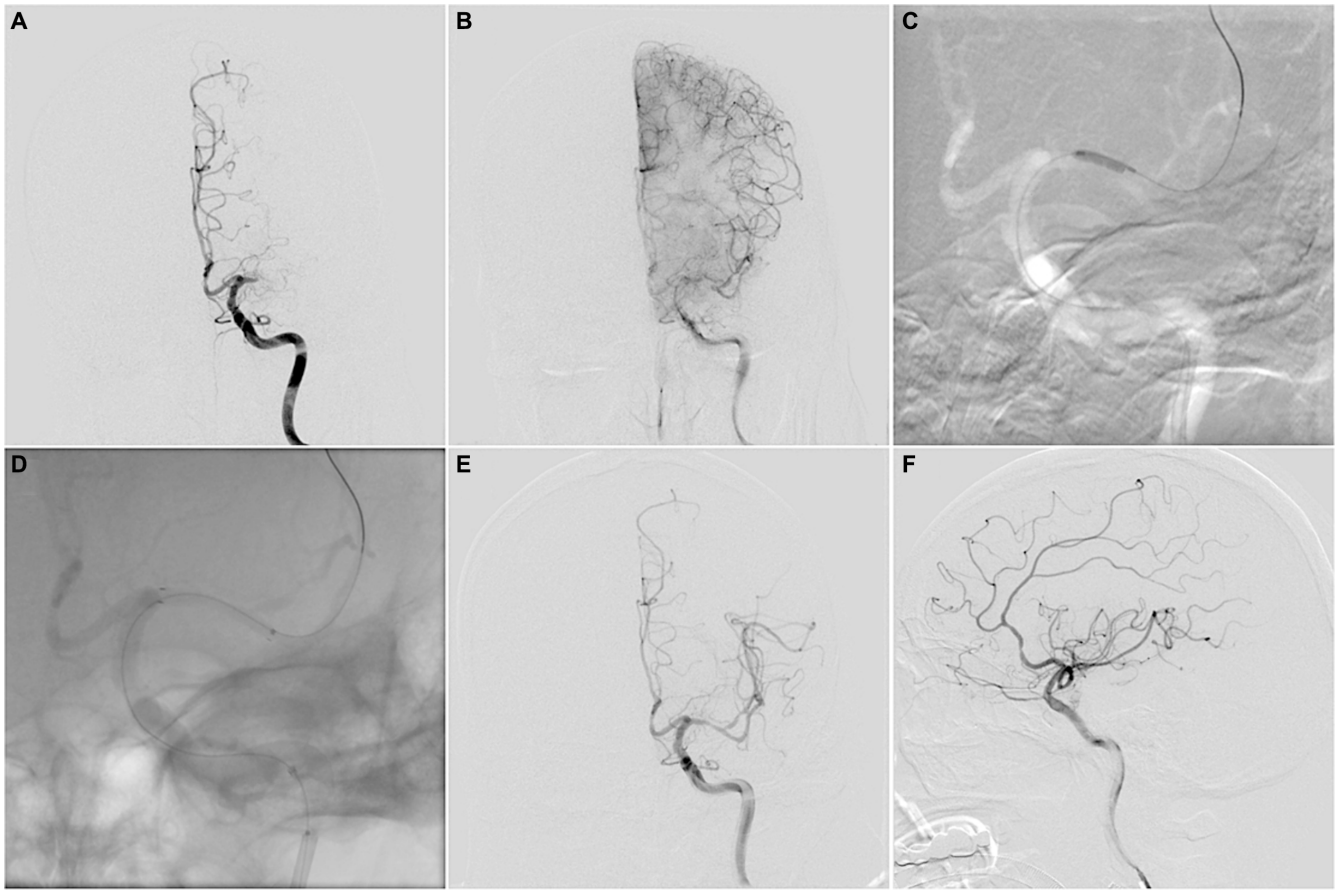
**(A,B)** MCAO-SEED scoring before endovascular recanalization: a “tapered” MCA stump (0), occlusion length ≤ 10 mm (0), bending ≥90° (0), a good vascular bed (0). **(C)** A Gateway angioplasty balloon was transported to the occluded lesion along the exchange microwire for dilation. **(D)** A Wingspan stent was deployed at the occluded lesion along the exchange microwire. **(E,F)** Successful recanalization was achieved for MCA, with a Thrombolysis in Cerebral Infarction (TICI) grade 3 score.

All operations were performed by experienced neurointerventional doctors (Endovascular treatment for more than 100 cases of acute intracranial large vessel occlusion and more than 50 cases of symptomatic MCA stenosis were completed). After general anesthesia, heparin was administered intravenously to keep the patient’s activated clotting time between 200 and 300 s. A microcatheter (Echelon-10, ev3 Neurovascular, Irvine, CA) was carefully traversed through the occluded segment under a microwire’s (Traxcess 014, MicroVention, Aliso Viejo, CA) guidance to the main M2 trunk. The operation was terminated if the microwire and microcatheter failed to cross the occluded segment and did not enter the true distal lumen. Then, an exchange microwire (Transend ES 014/300 Floppy, Boston Scientific Corp, Natick, MA) was fixed in an appropriate position, and the microcatheter was withdrawn. A 1.5 mm Gateway angioplasty balloon (Stryker Neurovascular, Fremont, CA) was transported to the occluded lesion along the exchange microwire and was slowly inflated to 6–10 atmospheres at a rate of 30 s per atmosphere. In case of excess local residual stenosis (>50%), a second balloon dilation was performed or another Gateway balloon with 2 mm diameter was used for dilation. A self-expandable stent (Wingspan stent, Stryker Neurovascular, Fremont, CA; Solitaire, Stryker Neurovascular, Fremont, CA; or Enterprise, Codman & Shurtleff, Raynham, MA) was placed at the occluded lesion as per the operator’s preference. Subsequently, a postoperative angiography confirmed the patency. A DynaCT was performed immediately after the operation (Figure 2).

### 2.4 Peri-procedural medical management

The periprocedural protocols were standardized across the four hospitals where the research was conducted. Before the procedure, the patients were given daily doses of aspirin (100 mg) and clopidogrel (75 mg) for 1 week; thromboelastography platelet mapping was analyzed to guide the antiplatelet treatment. In case of aspirin resistance, the daily aspirin dose was increased (200 or 300 mg). If clopidogrel resistance existed, the patients were administered ticagrelor 90 mg twice a day. Moreover, dual antiplatelet therapy was given for 3 months, followed by lifelong aspirin or clopidogrel monotherapy. Intensive statin treatment was started a week preoperatively and was continued for 1 month, with a lifelong low statin dose for maintenance. Postoperatively, the patient’s blood pressure was maintained at 100–130/60–80 mm Hg to reduce the reperfusion hemorrhage risk. Furthermore, the patients were also educated regarding the ways to control other risk factors.

### 2.5 Follow-up management and data collection

The protocols for the CT and MRI scans were standardized across the hospitals involved, and those who reported these scans were physician-in-charge or deputy chief physician. A brain computed tomography (CT) scan identified potential intracranial hemorrhages (ICH) 24 h and 7 days postoperatively. An MRI scan was consequently performed to identify any new cerebral infarctions 3 days postoperatively. Additionally, MRA or CTA was done to determine the recanalization of the occluded MCA 3 days postoperatively. The scores of mRS and the National Institutes of Health Stroke Scale (NIHSS) were obtained at the admission time, preoperatively, 24 h post-procedure, at the time of discharge, and 30 days postoperatively.

The demographic, clinical, procedural, imaging, and follow-up data were collected. Successful recanalization was defined as an antegrade flow with a modified Thrombolysis in Cerebral Infarction (TICI) grade ≥ 2b and a residual stenosis of ≤50% postoperatively. However, the periprocedural complications included ICH, hyperperfusion syndrome, vessel perforation, perforating branch occlusion, MCA branch embolization, dissection, and death within 30 days of the procedure. Additionally, the successful microwire crossing time (TMO) was designated as a parameter to evaluate the technical drawbacks.

### 2.6 Statistical analysis

Statistical analyses were performed with SPSS 25.0 software (IBM, Armonk, NY, United States). The baseline data, perioperative results, and clinical and imaging follow-up data were compared within all MCAO-SEED scores. All normally distributed continuous and quantitative variables were expressed as the mean ± SD while non-normally distributed and categorical variables were expressed as the median as well as IQR and proportions, respectively. Intragroup comparisons were performed using Kruskal–Wallis and χ2 tests for continuous and categorical variables, respectively. The receiver operating characteristic (ROC) curve was used to identify the optimal cut-off point for determining successful recanalization. Additionally, a multivariate analysis using a logistic regression model was conducted to determine positive predictors for successful recanalization. All statistical tests were two-sided, and *p*-values <0.05 were considered statistically significant.

## 3 Results

### 3.1 Baseline characteristics

A total of 113 patients comprising 79 men and 34 women with symptomatic non-acute MCA occlusion were enrolled. They all underwent a cerebral angiography assessment and MCAO-SEED scoring before endovascular recanalization. The median time from the last symptom onset to the endovascular treatment was 26.0 days (IQR 17.0–37.0). No significant difference was observed in the baseline characteristics between the patients with different MCAO-SEED scores. The detailed baseline data is provided in Table 1.

**Table 1:** Baseline characteristics.

Characteristics	Overall (*n* = 113)	0 (*n* = 32)	1 (*n* = 40)	2 (*n* = 16)	3 (*n* = 19)	4 (*n* = 6)	*p*
Age (years), median (IQR)	61 (52, 57)	61 (51.5, 68.75)	59 (47.75, 70.75)	62.5 (52.25, 67)	64 (57, 67)	61.5 (53.5, 68.75)	0.309
Male sex, no. (%)	79 (69.9)	22 (68.8)	27 (67.5)	11 (68.8)	14 (73.7)	5 (83.3)	0.973
Risk factors, no. (%)							
Hypertension	87 (77)	25 (78.1)	31 (77.5)	9 (56.3)	17 (89.5)	5 (53.3)	0.25
Diabetes Mellitus	30 (26.5)	12 (37.5)	6 (15)	4 (25)	7 (36.8)	1 (16.7)	0.182
Dyslipidemia	59 (52.2)	12 (37.5)	26 (65)	9 (56.3)	7 (36.8)	5 (83.3)	0.054
Cardiac disease	7 (6.2)	1 (3.1)	4 (10)	0 (0)	2 (10.5)	0 (0)	0.59
Smoking	41 (36.3)	10 (31.3)	15 (37.5)	5 (31.3)	10 (52.6)	1 (16.7)	0.494
History of ischemic stroke	36 (31.9)	8 (25)	13 (32.5)	4 (25)	8 (42.1)	3 (50)	0.582
Qualifying events, no. (%)							
Recent stroke	87 (77)	27 (84.4)	32 (80)	8 (50)	16 (84.2)	4 (66.7)	0.081
Recent TIA	19 (16.8)	4 (12.5)	5 (12.5)	5 (31.3)	4 (21.1)	1 (16.7)	0.42
Last symptom to recanalization (days), median (IQR)	26 (17, 37)	24 (17, 36.75)	25 (14.75, 37)	33 (20, 35.75)	36 (25, 41)	22 (15.25, 26)	0.064
MRS before onset of Symptom, median (IQR)	0 (0, 0)	0 (0, 0)	0 (0, 0)	0 (0, 0)	0 (0, 0)	0 (0, 0.25)	0.489
Preoperative NHISS, median (IQR)	2 (0,5)	2 (0, 4)	3 (1.25, 6)	1 (0, 3)	2 (1, 5)	0 (0, 3)	0.052
Preoperative MRS, median (IQR)	1 (1, 3)	1 (1, 3)	2 (1, 3)	1 (0.25, 2)	1 (1, 2)	1 (0, 1.75)	0.365

IQR interquartile range, TIA Transient Ischemic Attacks, NIHSS National Institutes of Health stroke scale score, MRS modified Rankin Scale.

### 3.2 Perioperative outcomes and preliminary clinical results

The total successful recanalization and perioperative complication rates were 83.2% (94/113) and 15.9% (18/113), respectively. Moreover, 65.5% (74/113) of them underwent stent implantation. The detailed clinical and angiographic outcomes are shown in Table 2. 96.9, 90, 87.5, 52.6, and 50% of the patients achieved recanalization with MCAO-SEED scores of 0, 1, 2, 3, and 4 (*p*<0.001), respectively. Conversely, the perioperative complication rates were (3.1% vs. 7.5% vs. 6.3% vs. 52.6% vs. 50.0%; *p* < 0.001). The median TMO in the MCAO-SEED score of 0 group was shorter than the other groups (2 min, 9 min, 8.5 min, 14 min, and 20 min; *p* < 0.001).

**Table 2:** Perioperative outcomes and preliminary clinical results.

Characteristics	Overall (*n* = 113)	0 (*n* = 32)	1 (*n* = 40)	2 (*n* = 16)	3 (*n* = 19)	4 (*n* = 6)	*p*
With stent implantation, No. (%)	74 (65.5)	24 (75)	26 (65)	11 (68.8)	10 (52.6)	3 (50)	0.103
Technical success, no. (%)	94 (83.2)	31 (96.9)	36 (90)	14 (87.5)	10 (52.6)	3 (50)	**<0.001**
TMB (min), median (IQR)	6 (2.25, 10.75)	2 (1, 4)	9 (5, 11)	8.5 (3.75, 11.5)	14 (6, 18)	20 (17.5, 24)	**<0.001**
Periprocedural complications, no. (%)	18 (15.9)	1 (3.1)	3 (7.5)	1 (6.3)	10 (52.6)	3 (50)	**<0.001**
Perforated bleeding, no. (%)	5 (4.4)	0 (0)	0 (0)	1 (6.3)	4 (21.1)	0 (0)	**0.004**
Dissection, no. (%)	2 (1.8)	0 (0)	0 (0)	0 (0)	2 (10.5)	0 (0)	0.076
Reperfusion hemorrhage, no. (%)	3 (2.7)	0 (0)	1 (2.5)	0 (0)	0 (0)	2 (33.3)	0.054
Thrombosis, no. (%)	4 (3.5)	1 (3.1)	1 (2.5)	0 (0)	2 (10.5)	0 (0)	0.585
Perforator infarction, no. (%)	4 (3.5)	0 (0)	1 (2.5)	0 (0)	2 (10.5)	1 (16.7)	**0.024**
Death within 30 days, no. (%)	0 (0, 0)	0 (0, 0)	0 (0, 0)	0 (0, 0)	0 (0, 0)	0 (0, 0)	0.999
mRS score at 90 days, median (IQR)	1 (0, 2)	1 (0, 2)	1 (0, 2)	1 (0, 1.75)	1 (0, 2)	1.5 (0, 4)	0.957
mRS score before operation vs. mRS score at 90 days	1 (1, 3):1 (0, 2)						0.063

IQR interquartile range, mRS modified Rankin Scale, TMB time of successful microwire crossing the occlusion lesion.

### 3.3 MCAO-SEED score value for recanalization

The TMO was associated with the MCA occlusion length, a tapered stump, an MCA bend, and the distal vascular bed of the MCAO. However, the successful recanalization and perioperative complication rates were related to the MCA occlusion length, MCA bending, and the distal vascular bed of the MCAO but had nothing to do with a tapered stump (Table 3).

**Table 3:** The analysis of grading difficulty of MCAO-SEED scoring.

Characteristics	Technical success, no. (%)	TMB (min), median (IQR)	Periprocedural complications, no. (%)
Block section>10 mm (*n* = 58)	43 (74.1)	10 (6, 14)	15 (25.9)
Block section≤10 mm (*n* = 55)	51 (92.7)	3 (2, 8)	3 (5.5)
*p*	**0.008**	**<0.001**	**0.003**
Nontapered (*n* = 29)	22 (75.9)	10 (6, 16.5)	6 (20.7)
Tapered (*n* = 84)	72 (85.7)	5 (2, 10)	12 (14.3)
*p*	0.35	**0.001**	0.604
No distal collateral retrograde filling (*n* = 24)	12 (50)	14 (6, 20)	13 (54.2)
Distal collateral retrograde filling (*n* = 89)	82 (92.1)	6 (2, 10)	5 (5.6)
*p*	**<0.001**	**0.002**	**<0.001**
Angle<90° (*n* = 42)	29 (69)	10.5 (5.75, 15)	13 (31)
Angle≥90° (*n* = 71)	65 (91.5)	5 (2, 9)	5 (7)
*p*	**0.002**	**<0.001**	**0.001**

IQR interquartile range, mRS modified Rankin Scale, TMB time of microguide wire through the block.

### 3.4 MCAO-SEED score ≤ 2 was an independent predictor for successful recanalization

**Figure 3:**
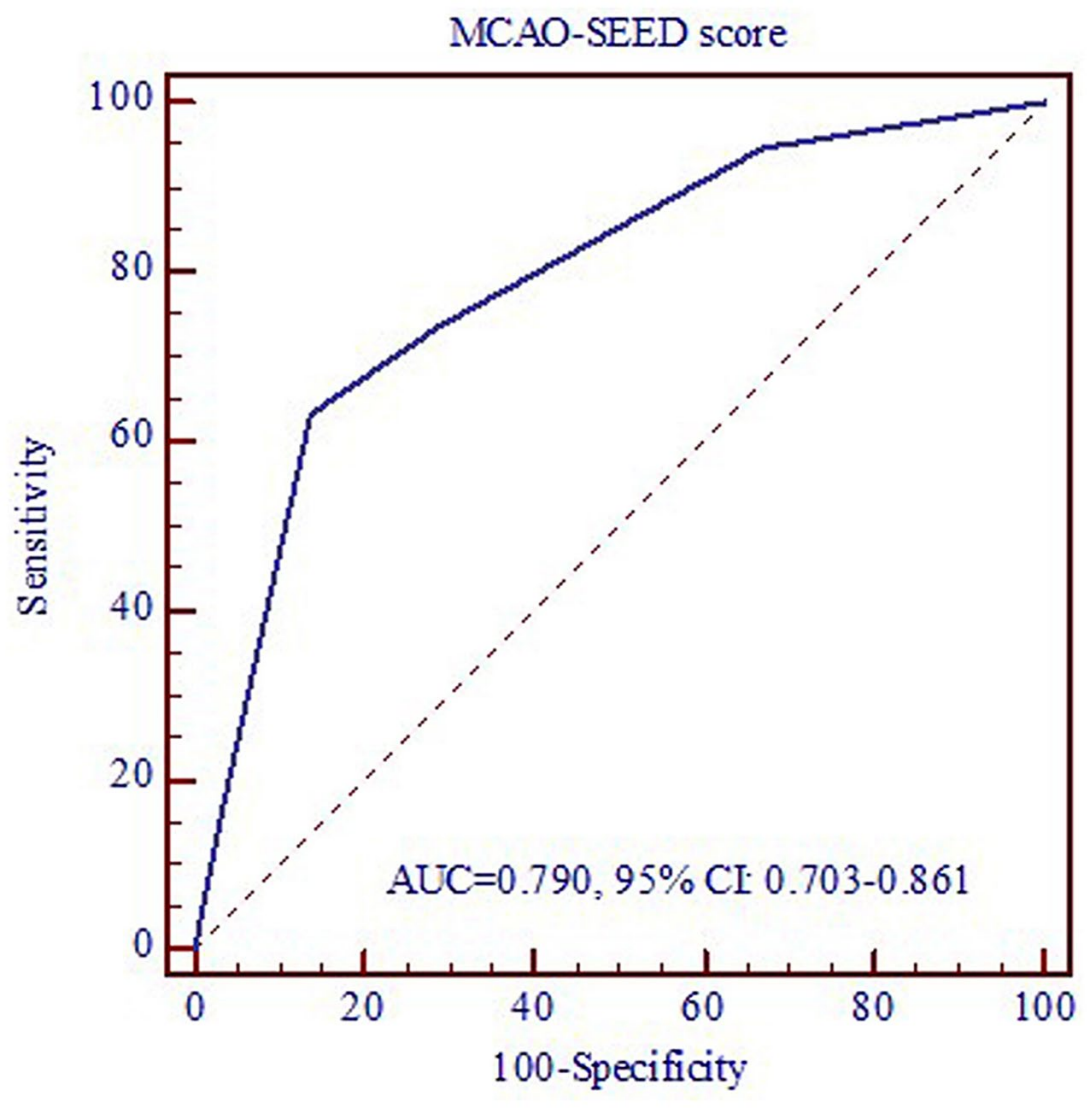
ROC curve of MCAO-SEED score in determining successful recanalization.

The ROC curve was used to evaluate the predictive value of the MCAO-SEED recanalization score (Figure 3). The area under the ROC curve (AUC) was 0.790 (0.703–0.861), suggesting a well-predictive performance of the MCAO-SEED score. We also found that when the optimal cut-off point of the MCAO-SEED score was 2, the sensitivity and specificity were 86.2 and 63.2%, respectively. The patients with an MCAO-SEED score ≤ 2 displayed a higher successful recanalization rate, a shorter TMO, and a lower perioperative complication rate than the patients with an MCAO-SEED score>2 (Table 4). However, no significant difference was observed in the baseline characteristics between the two groups (Supplementary Table S1). Thus, an MCAO-SEED score ≤ 2 and the patient’s age were the two independent predictors for successful recanalization (Table 5).

**Table 4:** Perioperative outcomes and preliminary clinical results according to MCAO-SEED score.

	MCAO-SEED score		
	≤2	>2	*P*
With stent implantation, No. (%)	61 (69.3)	13 (52.0)	0.108
Technical success, no. (%)	81 (92.1)	13 (52.0)	<0.001
TMB (min), median (IQR)	5 (2, 9)	15 (10, 20)	<0.001
Periprocedural complications, no. (%)	5 (5.7)	13 (52.0)	<0.001
Perforated bleeding, no. (%)	1 (1.1)	4 (16.0)	0.008
Dissection, no. (%)	0 (0.0)	2 (8.0)	0.047
Reperfusion hemorrhage, no. (%)	1 (1.1)	2 (8.0)	0.123
Thrombosis, no. (%)	1 (1.1)	3 (12.0)	0.033
Perforator infarction, no. (%)	2 (2.3)	2 (8.0)	0.212
Death within 30 days, no. (%)	0 (0, 0)	0 (0, 0)	-
mRS score at 90 days, median (IQR)	1 (0, 2)	1 (0, 2)	0.857

**Table 5:** Multivariable logistic regression analysis for successful recanalization.

	OR (95% CI)	*P*
MCAO-SEED score **≤** 2	26.13 (5.69–120.05)	<0.001
Age (per 10 yrs)	0.51 (0.26–0.98)	0.044
Male sex	0.32 (0.06–1.72)	0.182
Smoking	2.40 (0.50–11.46)	0.274
Hypertension	1.81 (0.34–9.79)	0.490
Diabetes Mellitus	0.55 (0.10–3.05)	0.495
Dyslipidemia	1.04 (0.26–4.14)	0.951
History of ischemic stroke	0.17 (0.03–1.04)	0.056
MRS before onset of Symptom	2.01 (0.63–6.46)	0.242
Preoperative NHISS	0.91 (0.69–1.21)	0.517

## 4 Discussion

Despite AMT, treating recurrent symptomatic non-acute MCA occlusion remains a challenge. The efficacy of EC-IC arterial bypass surgery is yet to be demonstrated in larger studies, even in hypoperfusion patients (Bambakidis and Chowdhry, 2010). However, a successful recanalization of a non-acute MCAO patient might improve the patient’s cognitive function, attention, and psychomotor processing speed. Recently, some clinical studies have reported the feasibility of endovascular recanalization for these patients.

However, many doctors do not attempt recanalization in these patients owing to higher technical difficulty, lower successful recanalization rate, and more perioperative complications. These factors are closely related to the different preoperative evaluation and selection criteria of patients. Gao et al.^12^ indicated that patients with a classification Type I MCA occlusion displayed a higher technical success rate (95.5%) and a lower perioperative complication rate (4.5%), which might be attributed to better preoperative assessment. However, this classification system might be too simple because preoperative angiography can often provide more clinical data and predictions. Based on several cases in recent years, we established an MCAO-SEED scoring to provide a good evaluation tool for clinicians to predict successful recanalization as well as technical and perioperative complications of interventional recanalization. After verifying the validity of the scoring system through a double-blind method, we found that a lower MCAO-SEED score was associated with a higher successful recanalization rate, a shorter TMO, and a lower perioperative complication rate. Furthermore, when the optimal cut-off point of the MCAO-SEED score was 2, the sensitivity and specificity were 86.2 and 63.2%, respectively.

A tapered MCA stump guides the microwire easily to enter the MCA occlusive segment (Chen et al., 2016), increases its stability in the MCA, and improves the possibility of discovering the true MCA lumen. Additionally, a tapered stump could also help in the proper placement of the microcatheter at the proximal end of the occluded MCA and transmission of the microwire’s radial forces into the distal lumen. Subsequently, it reduced the incidence of adverse events of perforation and rupture. Our study indicated that the patients with a tapered stump had reduced TMO (2.5 min) and perioperative complication rate (5%) than the patients with a blunt stump (10 min; 36%). However, no significant difference was observed in the successful recanalization rate between the two groups (82%, 41/50; vs. 66.7%, 10/15).

The occlusal length is a key factor for MCA recanalization. Moreover, the occlusion length affected the successful recanalization rate of chronic total coronary artery occlusion (CTO) (Morino et al., 2011). A longer occlusion length was also associated with a reduced successful recanalization rate, as suggested by Gao et al. (2021). In our study, a length < 10 mm was equivalent to the type B stenotic lesion length as per the Mori classification (Mori et al., 1998). Thus, a longer occlusion length predicted a more variable artery course, an easier entry for false lumen, and a higher incidence of arterial injury and perforation rupture, thereby resulting in an enhanced technical difficulty rate and a lower successful recanalization rate. In our study, 97.5% (39/40) of the MCAO patients with length ≤ 10 mm had successful recanalization which was higher than the patients with a length > 10 mm (48%, 12/25). Hence, the patients with lengths ≤10 mm had reduced TMOs and perioperative complication rates.

MCA bending and the distal vascular bed of MCAO were the other two main key components that contributed to the technical drawbacks and successful recanalization rate. The MCA bending was described in type C stenotic lesions as per the Mori classification. As reported by Mori et al. (1998), extreme angulation was a prime reason for the failure of MCA recanalization. It was suggested that if the angle was ≥90° between the MCA segment and the main MCA trunk, the microguide wire’s radial force transmission was better and led to the stability of the microcatheter when the microguide wire crossed the occlusion lesion reaching the M2 segment. The microguide wire and microcatheter were easier to place and provided exchange at the M2 segment at the distal end, leading to a shorter successful microwire crossing time and reduced vessel perforation rate. In our study, the TMO of the patients with an angle ≥90° (3 min) was significantly shorter than the patients having an angle <90° (10 min). Additionally, a higher incidence of successful recanalization (89%, 33/37) and fewer perioperative complications (5.4%, 2/37) occurred in patients with angles ≥90° than in the patients with angles <90° (64.3%, 18/28; 32.1%, 9/28). Meanwhile, the distal vascular bed was a manifestation of the collateral circulation and perfusion at the distal end of the MCAO. Occlusion involving bifurcations is a negative predictor for technical success in endovascular recanalization for chronic CTO (Stone et al., 2005; Galassi et al., 2015) and MCAO (Gao et al., 2021). A good vascular bed provides good distal vessel visibility, allows better navigation for microguide wire, reduces the chances of the microguide wire entering the false lumen, and decreases the incidence of hyperperfusion syndrome. We also suggested that the patients with a good vascular bed displayed more successful recanalization (89.8%, 44/49) and a lower probability (6.1%, 3/49), while the TMO (3 min) decreased for the patients with good vascular beds.

This study had a few limitations. First, the retrospective design might have induced patient selection and recall biases that may have affected the results. Therefore, the results need to be demonstrated by subsequent prospective large-sample studies. Second, the study lacked 90-day or longer follow-up results, such as the MCA restenosis rate and stroke recurrence. However, the MCAO-SEED scoring was mainly designed to predict technical success.

In conclusion, the MCAO-SEED scoring can effectively predict the successful recanalization rate, perioperative complication rate, and the TMO of endovascular recanalization for non-acute MCAO and may also be used to grade the difficulties encountered during the surgery. The MCAO-SEED score can also provide surgeons with a better preoperative evaluation for treating symptomatic non-acute MCAO patients and can help in choosing suitable patients, especially patients with MCAO-SEED score ≤ 2. Thus, our results need to be further validated by subsequent prospective large-sample studies.

## Data Availability

The raw data supporting the conclusions of this article will be made available by the authors, without undue reservation.
