# Supplementary material for: A new angiographic scoring for grading the difficulty of recanalization for symptomatic non-acute middle cerebral artery occlusions
Source: Front Neurosci. 2024 Oct 8;18:1398749. doi: 10.3389/fnins.2024.1398749 (PMC11493690; doi:10.3389/fnins.2024.1398749)
Supplement: Supplementary file 1 [file Table_1.DOCX]

Supplementary Material

# Supplementary Table

**Table S1.** Baseline characteristics of study participants according to MCAO-SEED score.

|  | **MCAO-SEED score** | |  |
| --- | --- | --- | --- |
|  | **≤2** | **>2** | ***P*** |
| Participants, No | **88** | **25** |  |
| Age (years), median (IQR) | 61(51, 68) | 64(56, 67) | 0.404 |
| Male sex, no. (%) | 60(68.2) | 19(76.0) | 0.452 |
| Risk factors, no. (%) |  |  |  |
| Hypertension | 65(73.9) | 22(88.0) | 0.138 |
| Diabetes Mellitus | 22(25.0) | 8(32.0) | 0.484 |
| Dyslipidemia | 47(53.4) | 12(48.0) | 0.633 |
| Cardiac disease | 5(5.7) | 2(8.0) | 0.649 |
| Smoking | 30(34.1) | 11(44.0) | 0.363 |
| History of ischemic stroke | 25(28.4) | 11(44.0) | 0.140 |
| Qualifying events, no. (%) |  |  |  |
| Recent stroke | 67(76.1) | 20(80.0) | 0.685 |
| Recent TIA | 14(15.9) | 5(20.0) | 0.629 |
| Last symptom to recanalization (days), median (IQR) | 25 (17, 37) | 32 (23, 38) | 0.114 |
| MRS before onset of Symptom, median (IQR) | 0 (0, 0) | 0 (0, 0) | 0.957 |
| Preoperative NHISS, median (IQR) | 2 (0, 5) | 2 (0, 3) | 0.551 |
| Preoperative MRS, median (IQR) | 1 (1, 3) | 1 (1, 2) | 0.353 |
